# Lower Respiratory Tract Infection Trends in East and South-East Asia: In the Light of Economic and Health Care Development

**DOI:** 10.1177/2333794X21989530

**Published:** 2021-01-24

**Authors:** Jelle J. Feddema, Anne M. van der Geest, Eric Claassen, Linda H. M. van de Burgwal

**Affiliations:** 1 Vrije Universiteit Amsterdam, Amsterdam, Noord-Holland, The Netherlands

**Keywords:** respiratory tract infection, mortality, health access, healthcare quality, epidemiology, economic growth, South-East Asia

## Abstract

This study explored to what degree economic development and improvement of healthcare are associated with lower respiratory tract infection (LRTI) mortality. A correlation analysis between LRTI mortality and Gross Domestic Product (GDP) per capita, and the Health Access and Quality Index (HAQI), respectively was conducted for 15 countries in East and South-East Asia. The results revealed a dramatic decrease in LRTI mortality in total populations for lower-middle income (LMI) countries but at the same time an increase in upper-middle income (UMI) and high-income (HI) countries. A highly significant (*P* < .001) growth-dependent relationship between LRTI mortality and economic growth was observed. Improvements in HAQI were significantly associated with a decrease in LRTI mortality in LMI countries, but an increase in UMI and HI countries. The decline of LRTI mortality amongst children in LMI countries is an encouraging trend and efforts against LRTI must be continued, though not at the expense of preparing health systems for the growing burden.

## Introduction

The burden of lower respiratory tract infections (LRTI) has declined substantially globally over the last decades.^1^ International efforts to combat respiratory infections such as the Millennium Development Goals (MDG) have been successful in reducing the disease burden, though reduction appears to be inconsistent across locations and countries. In some developing countries the rate of mortality decline is far higher than target rates set by international goals whereas in other developing countries the rate of decline is much lower.^1^ The overall decrease in childhood pneumonia mortality is an encouraging trend, though infections of the respiratory tract remain the leading cause of mortality under 5 years old. In developing countries, more than 10 million children die before the age of 5 of which a third can be attributed to LRTI.^2^ It is in these low-resource settings where 81% of all LRTI related deaths in children occur, thereby remaining the leading cause of children’s mortality and hospitalizations.^3^ So while some progress has been made in reducing the LRTI-related disease burden, these infections continue to pose an immense burden on healthcare systems leading to many deaths of which the majority seems avertable.

Lifestyle factors such as indoor air pollution, living in a large household, having regular contact with children and being underweight are all associated with childhood LRTI.^4,5^ Being underweight appears to be particularly relevant for low income countries where mortality due to respiratory infections is high. Malnutrition predisposes young children to pneumonia due to its negative consequences on their immune system.^6^ However, while living conditions such as poverty and malnutrition underlie the high incidence of pneumonia in developing regions, poor access to healthcare services and the absence of LRTI treatments are the main cause of high mortality rates.^4^ Also, parents often lack adequate knowledge on crucial symptoms and do not perceive the illness as serious or life threatening for their children, despite the fact that relatively inexpensive and effective measures are available for prevention and treatment of these infections.^7^

The impact of economic conditions on public health and the importance of economic growth in reducing infectious disease mortality have long been recognized.^8,9^ Economic development is indirectly associated with respiratory disease burden as it improves the standard of living and well-being of the population, and increases governments’ investments into the healthcare sector.^10-12^ On the level of the individual, economic growth translates into improved socio-economic status (SES) and decreased exposure to risk factors as children living in poor economic household conditions are more likely to suffer from respiratory infections than children living in better households.^11,13^ On a system-wide level, economic growth stimulates healthcare expenditures and gives healthcare authorities the means to provide better access to, and higher quality of the public health system. Better access to health facilities appears to be particularly valuable considering its inverse relationship to the LRTI burden.^4^

Interestingly, in South East Asia (SEA) the respiratory burden remains amongst the highest worldwide and reduction here appears to be inconsistent despite improvements in living standards and healthcare systems.^1,14^ SEA has some of the highest rates of LRTI related mortality in children with numbers exceeding those of many other regions in the world.^15^ Ongoing measures seem insufficient and preventive interventions and management strategies do not seem to reach those who need it the most resulting in a disproportionally high burden compared to other parts of the world. It is important to gain a better understanding of how this burden is distributed among countries to evaluate strategies and implement effective measures in different healthcare systems. Therefore, this study explored how trends of respiratory burden in SEA have evolved over the years, and to what degree economic growth and development of healthcare systems correlate with the burden caused by these infections.

## Methods

Qualitative data from various publicly available databases was collected to assess the respiratory burden in 15 East and South-East Asian countries. Data was visualized in graphs and correlation analyses were conducted to determine the strength of associations between variables.

### Variables

Three variables were included in the analysis to provide a comprehensive overview of mortality trends related to lower respiratory tract infections. First of all, lower respiratory tract infection mortality measured in death rate per 100 000 population was chosen as an indicator for the respiratory disease burden. A distinction was made between LRTI mortality in all ages and in children <5 years old. Mortality numbers were retrieved from the Global Burden of Disease (GBD) database constructed by the Institute for Health Metrics and Evaluation (IHME).^16^

Secondly, economic development measured by growth in Gross Domestic Product (GDP) per capita (PPP) was selected as an indicator for an individual’s well-being and level of socio-economic status. Investigation of a previously published wealth to wellbeing coefficient showed that GDP is well correlated with the living standards of a population and improvements thereof should be accompanied with a decrease in LRTI related mortality.^16,17^

Lastly, the Health Access and Quality Index (HAQI) was included as an variable to estimate the access to and quality of healthcare. The index was calculated by measuring the number of deaths by 32 disease causes which should not occur in the presence of effective care.^18^ We hypothesized that improvement in health access and quality should be followed by a reduction in LRTI mortality. Yearly HAQI values were calculated based on the published 5-year HAQI values to increase the number of data points for the correlation analysis.

### Study Subjects

Data on LRTI mortality in East and South-East Asia from 2000 to 2017 was analyzed. Hong-Kong, Macau, and North-Korea were excluded since data concerning these countries was not readily available. As such, this research included the following countries: Brunei, Cambodia, China, Indonesia, Japan, Laos, Malaysia, Mongolia, Myanmar, Philippines, Singapore, South-Korea, Taiwan, Thailand, and Vietnam.

### Group Characteristics

Countries were divided into one of the following groups according to the World Bank Atlas categorization: LMI economies, UMI economies, and HI economies.^19^ The Supplemental Table 1 shows the classifications of East and South-East Asian countries according to the World Bank criteria based on their status in 2000.

### Correlation Analysis

Analyses were performed in Prism8. The Shapiro-Wilk test was used to test for normality amongst the variables. Subsequently, the Pearson correlation coefficient and statistical significance were calculated to measure the strength of the following suspected associations: LRTI mortality and GDP, and LRTI mortality and HAQI. In studying the correlation between LRTI mortality and GDP, the values of variables were expressed as percentages relative to the year 2000, allowing to design graphs with the same axis scales for developing and developed countries.

### Ethical Approval and Informed Consent

For this type of study formal consent or ethical approval was not required as no studies with animals or human participants were performed by any of the authors. All the data obtained in this study was available within the public domain.

## Results

The total number of countries included in this study was fifteen. Their Gross Domestic Product (GDP) per capita (PPP) ranged from $4.018 to $94.105 as showed in Supplemental Figure 1. Growth in GDP per capita in low-middle (133%-453%), and upper-middle (114%-430%) income countries has been achieved at a much faster rate compared to high income countries (19%-130%). Nevertheless, the absolute value of GDP for LMI and UMI countries remains lower compared to HI countries as can be seen in Supplemental Figure 2.

**Figure 1.**
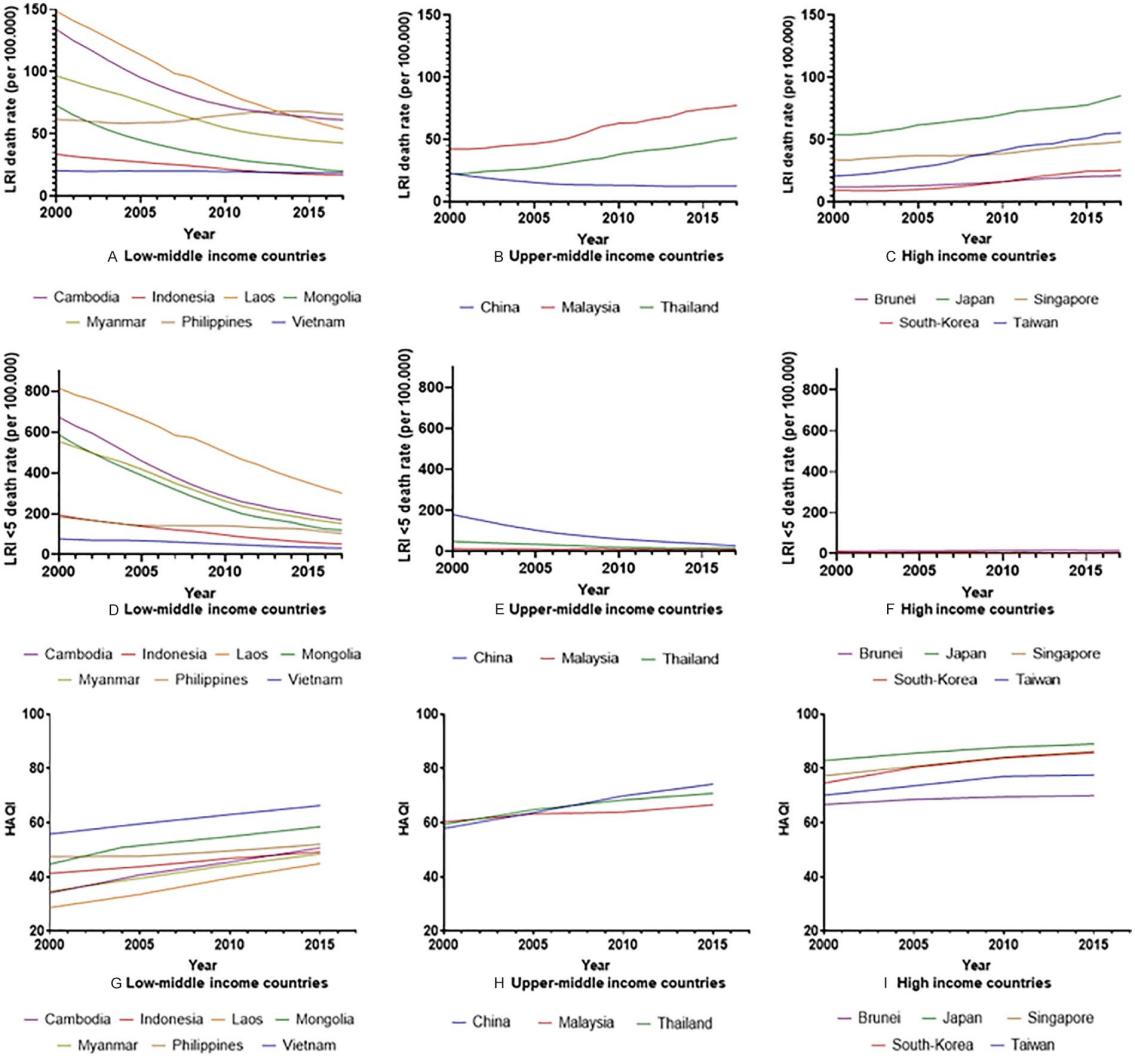
The lower respiratory infection (LRTI) mortality for all age groups has decreased in all low-middle income countries except the Philippines (A). An increase in LRTI mortality for all age groups was found in upper-middle and high income countries with the exception of China (B and C). The mortality of LRTIs amongst children <5 years has decreased for all countries except Brunei (D and F). The Health Access and Quality Index (HAQI) has increased for all countries (G and I).

Figure 1A to F display LRTI-related mortality trends for the general population (A–C) and in children <5 years (D-F), for low-middle income countries, upper-middle income countries, and high income countries. Figure 1A to C reveal opposite mortality trends within the same timeframe. In LMI countries, a substantial decrease in LRTI mortality per 100 000 population was seen with the exception of the Philippines where an increase in mortality from 61.57 to 65.73 per 100 000 is observable. The following changes in mortality were found for LMI countries: Cambodia 134.11 to 61.19 per 100 000, Indonesia; 33.80 to 16.95 per 100 000, Laos 148.67 to 53.89 per 100 000, Mongolia; 72.20 to 20.30 per 100 000, Myanmar; 96.68 to 42.77 per 100 000 and Vietnam; 20.41 to 18.86 per 100 000. Contrary to LMI countries, in most UMI and HI countries an increase in LRTI-related deaths was noticeable. The following changes in LRTI mortality were found for UMI and HI countries: China; 22.78 to 12.70 per 100 000, Malaysia; 42.35 to 77.33 per 100 000, Thailand; 22.38 to 51.24 per 100 000, Brunei; 12.02 to 21.13 per 100 000, Japan; 54.00 to 85.33 per 100 000, Singapore; 33.92 to 48.41 per 100 000, South-Korea; 9.50 to 25.52 per 100 000, and Taiwan; 20.86 to 55.61 per 100 000.

LRTI mortality in children <5 decreased in all countries except Brunei where it appeared to have grown from 12.80 to 15.17 per 100 000. The following changes in <5 mortality were found in HI and UMI countries: Japan; 5.14 to 2.62 per 100 000, Singapore 9.02 to 3.63 per 100 000, South-Korea; 5.46 to 1.49 per 100 000, Taiwan 5.31 to 4.96 per 100 000, China; 179.12 to 26.32 per 100 000, Malaysia; 13.26 to 9.02 per 100 000 and Thailand; 47.15 to 14.98 per 100 000. LMI countries displayed the highest reductions in <5 mortality where Cambodia showed a decrease from 673.75 to 169.64 per 100 000, Indonesia 194.19 to 51.89 per 100 000, Laos 815.02 to 300.48 per 100 000, Mongolia 587.08 to 119.68 per 100 000, Myanmar 555.30 to 152.58 per 100 000, the Philippines 183.03 to 103.81 per 100 000 and Vietnam from 76.17 to 31.30 per 100 000. While LMI showed a high decrease in <5 mortality, the mortality rates seen in UMI and HI countries were still considerably lower. Figure 1G to I shows that in line with expectations the HAQI has grown for all SEA countries in this sample and that the HAQI is generally seen higher in more developed countries.

**Figure 2.**
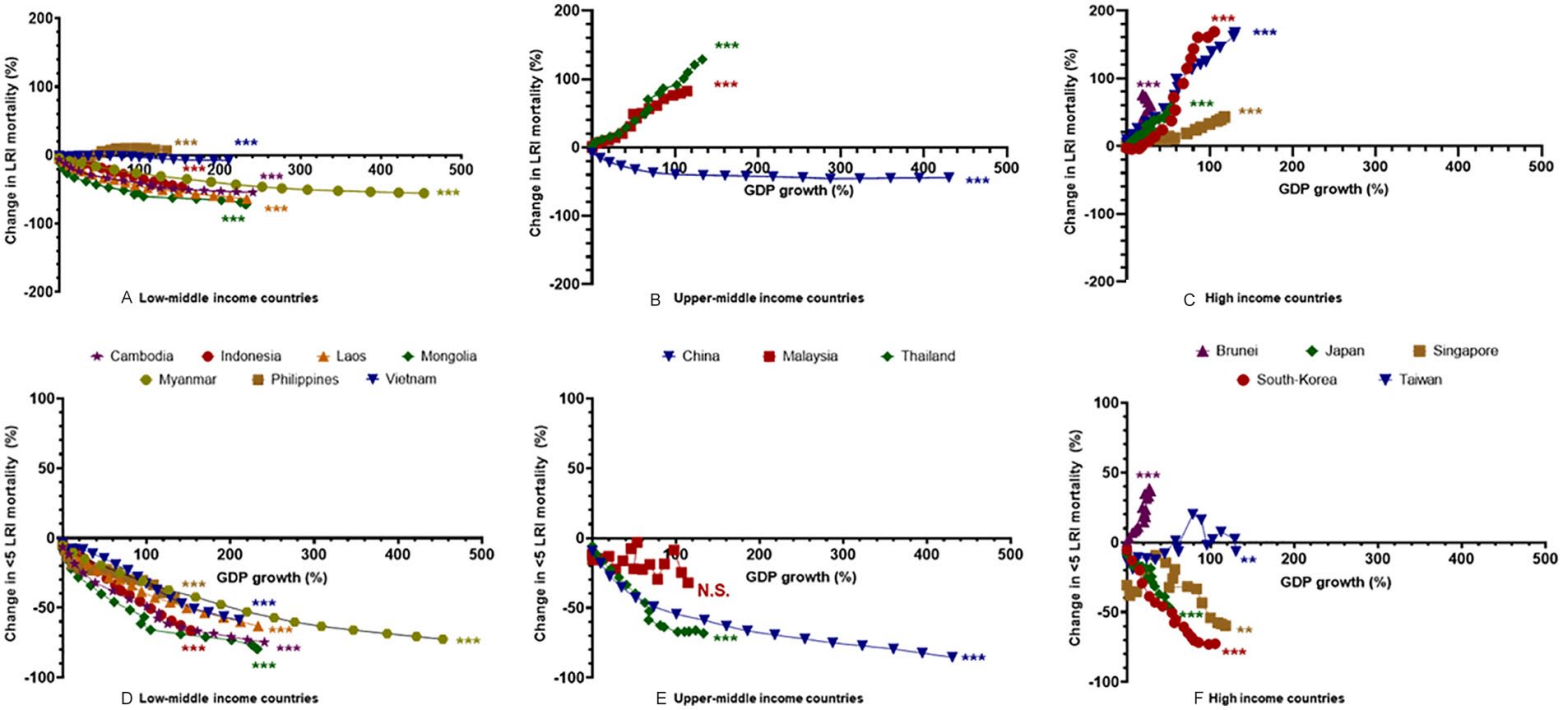
A strong significant correlation was found for all countries between Gross Domestic Product (GDP) per capita and LRTI mortality all ages (A-C). Countries characterized by a rapidly growing GDP revealed a negative correlation with LRTI mortality (A and B; Cambodia, Indonesia, Laos, Mongolia, Myanmar, Vietnam, China). Countries with a moderate growth in GDP revealed a positive correlation with LRTI mortality (B and C: Malaysia, Thailand, Brunei, Japan, Singapore, South-Korea, Taiwan). A significant correlation was also found between GDP and <5 LRTI mortality for all countries except Malaysia (D-F). These were all found to be negative correlations except for Brunei and Taiwan where a positive correlation was found showed a substantial growth in GDP that was correlated with a decrease LRTI mortality, whereas UMI countries and high income countries with a moderate growth in GDP correlated with an increase in LRTI mortality (A-C).

Figure 2 shows the correlation analysis for all countries between the variables GDP and LRTI mortality. Relative to the year 2000, LRTI mortality appeared to decrease up to 80% in LMI countries, whereas in UMI and HI countries an increase up to 170% was found. In various HI and UMI countries the LRTI mortality exceeded the mortality found in some of the LMI countries. The analysis revealed a very strong correlation (*P* < .001) for all countries between GDP growth (ppp, per capita) and LRTI mortality. HI countries plus Malaysia, Thailand and the Philippines, all characterized by a relatively moderate growth in GDP since 2000 varying between 19% and 133%, showed a positive correlation with LRTI mortality. All LMI countries (except the Philippines) and China, defined by a strong growth in GDP since 2000 varying between 152% and 453%, showed a negative correlation with LRTI mortality.

There also appeared to be a significant correlation between GDP growth and <5 LRTI mortality relative to 2000 for all countries except Malaysia. The correlation was negative in all cases indicating that <5 LRTI mortality decreased for all countries compared to the year 2000 regardless of how fast GDP developed.

**Figure 3.**
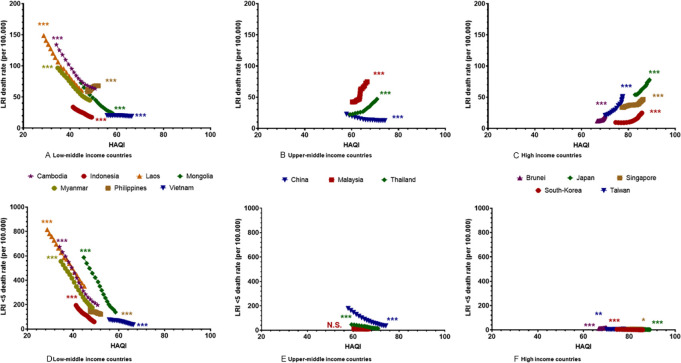
A strong significant correlation between the Health Access and Quality Index (HAQI) and LRTI mortality all ages was found for all countries (A-C). Improvements of the HAQI in low-middle income countries Cambodia, Indonesia, Laos, Mongolia, Myanmar Vietnam, and upper-middle country China were correlated with a decrease in LRTI mortality (A and B). Improvements of the HAQI for Malaysia, Thailand, Brunei, Japan, Singapore, South-Korea, and Taiwan were correlated with an increase in LRTI mortality (B and C). The correlation was also found for LRTI mortality <5 years (except Malaysia), where all countries revealed a negative correlation between HAQI development and LRTI <5 mortality except for Brunei which revealed a positive correlation (D and F).

Figure 3 displays the correlation between HAQI and LRTI mortality for LMI, UMI and HI countries. In most LMI countries and China, a significant negative correlation was observed indicating that improvement of health access and quality between the values of 30 and 70 correlated with a strong decrease in LRTI mortality. Contrary, in UMI countries Malaysia and Thailand, and all HI countries, a significant positive correlation indicated that an increase in HAQI between the values of 60 and 95 was associated with a rise in LRTI mortality.

A significant correlation between HAQI and <5 mortality was also found for all countries with the exception of Malaysia. In all cases the correlation coefficient was negative indicating that an increase in HAQI resulted in a decrease in <5 mortality. The only exception to this was Brunei and Taiwan where a positive correlation coefficient was found.

## Discussion

Here we present an overview of lower respiratory tract infections (LRTI) mortality trends in 15 South and South-East Asian countries (Brunei, Cambodia, China, Indonesia, Japan, Laos, Malaysia, Mongolia, Myanmar, Philippines, Singapore, South-Korea, Taiwan, Thailand, and Vietnam) over a 17-year period. We revealed a dramatic decrease in LRTI burden in low-middle income (LMI) but at the same time an increased respiratory burden in upper-middle (UMI) and high-income (HI) countries, where in some cases the mortality rate in HI and UMI countries exceeded that of LMI countries. Countries with a relatively moderate growth since 2000 revealed a steep increase in LRTI mortality rate, whereas countries characterized by a rapidly growing economy showed the opposite effect and displayed a fast decrease in LRTI burden. A correlation between the health and quality index (HAQI) was found in all LMI countries with improvement of the HAQI being associated with a strong decrease in LRTI mortality. The opposite correlation was found in UMI and HI countries where further improvements of the HAQI were associated with an increase in LRTI mortality.

LMI countries have been quite successful in reducing LRTI related death rates amongst their populations. At the same time, however, this study showed that the mortality in UMI and HI countries has been rising to such an extent that relatively more people die of respiratory infections in HI countries Japan, Singapore, and Taiwan compared to LMI countries Vietnam, Mongolia, and Indonesia. We hypothesize that differences in the composition of populations between HI and LMI- countries is the most likely reason why mortality rates in these developed regions are higher compared to some less developed areas.^18^ HI countries, and particularly Japan, Singapore, and Taiwan, all experience a rapidly aging population. This is considered the main cause of their rising mortality as pneumonia is a disease of the elderly.^20^ In LMI-countries, where life expectancy is much lower, the aging population is not the main contributor to LRTI mortality. Instead, exposure to key risk factors such as malnutrition and smoke pollution, and the absence of effective preventive measures seems to drive LRTI mortality in these countries.^20^ The reduction of the LRTI burden in these developing regions is therefore thought to be a result of both a decrease in incidence due to socioeconomic development and improved living standards, as well as improved access and quality of care.^21^

This study also confirmed that significant improvements in reducing <5 child mortality due to LRTI have been achieved in most countries. Especially in the LMI and UMI countries China, Cambodia, Indonesia, Laos, Mongolia, and Myanmar where <5 mortality was often reduced by a factor 5 in 2017 compared to 2000. Though, the progress that has been made countries is encouraging, the mortality rates are still high varying from 20 to 300 deaths per 100 000 children. Compared to HI countries, where children’s mortality due to LRTI is often lower than 5 per 100 000, the numbers in LMI countries highlight the importance of ongoing measures to further reduce LRTI mortality. Tackling key risk factors such as malnutrition and poor sanitation should remain important priorities in these LMI regions as they continue to pose problems despite best efforts.^22^ Additionally, socioeconomic risk factors such as a mother’s lack of education and inexperience should be targeted by public health interventions as it appears that they are associated with both the incidence and outcome of LRTI in children.^4^

Socioeconomic development and economic growth have both been associated with improvements in public health and have been found to be predictors of changes disease mortality.^23,24^ Our results indeed confirmed a relationship between economic growth and changes in LRTI mortality in which the level of economic growth determined the direction of this relationship. Countries with rapidly developing economies and GDP (152%-453%) revealed an inverse relationship with LRTI mortality, as hypothesized and published by earlier studies.^24^ This suggests that in these countries, rapid development of economies translated directly into health benefits for citizens, for instance by means of improved living standards. Countries characterized by a moderate growth in GDP (19%-133%), however, showed a positive relationship and rise in mortality. It appeared that in these regions the health benefits have already been gained in earlier development stages and are now outweighed by prosperity factors such as an aging population. An alternative explanation could be that economic surplus is no longer invested in healthcare improvements.

The growth-dependent relationship between economic development and public health has previously been studied where it was established that the effects of GDP growth on health outcomes, especially when measured by mortality rates, varies depending on a country’s stage of development.^12^ The effect of GDP growth on LRTI mortality appeared to be pro-cyclical in developing LMI countries but counter-cyclical in developed HI countries. In LMI countries, increases in GDP are often accompanied by reductions in death rates which is likely due to a higher consumption of food and increased spending on public health services. In HI countries, however, such growth in GDP often leads to unexpected increases in mortality rates caused by changes in health behavior and diseases linked with the prosperity of these populations. This opposing effect of economic growth on public health was also evident in this study where opposite LRTI trends were noticeable between LMI and HI countries.

Besides economic-growth dependent changes in LRTI mortality, this study also found changes in mortality dependent on the development of the HAQI. Amongst LMI countries, improvements in healthcare access and quality correlated with a decrease in LRTI mortality whereas in UMI and HI countries such improvements were not associated with lower mortality. Looking at graphs 3a to 3c there appears to be a ‘‘tipping point’’ around a HAQI of 60; a point on the HAQI scale where further improvement of healthcare services are no longer associated with decreased LRTI mortality. A possible explanation for this tipping point is to be found within the composition of the HAQ Index. The HAQ Index is comprised of a variety of communicable and non-communicable diseases that represent a range of health service areas and are considered amendable in the presence of effective care. Therefore, HAQI improvements in LMI countries must have been primarily the result of reducing amendable deaths of communicable diseases such as LRTIs through interventions such as vaccination, early diagnosis, and antibiotic treatment.^18^ HAQI improvements in UMI and HI countries, however, must have been achieved by reducing amenable deaths related to other diseases than respiratory infections seeing as LRTI mortality in these regions has been on the rise. These developed countries appear to have reached a point on the HAQI scale where further reduction of amenable deaths is not merely achieved by prevention and treatment of communicable diseases. Instead, improvement of healthcare quality and access requires a shift in focus toward non-communicable diseases. This importance of, and potential for improving non-communicable disease prevention and treatment has already been demonstrated in Europe and Central Asia where various countries saw significant HAQI gains in the last decade.^25^

Overall, the significant decline of LRTI mortality in developing nations is an encouraging trend and efforts against respiratory infections must be sustained going forward, though not at the expense of preparing health systems for the next generations. Already we see that LRTI mortality rates in some developed regions exceed those of developing regions, and with the projected growth in the elderly population this will only become more common. Recent demographic and epidemiologic trends indicate that by 2050, 2 billion of the world’s population will be older than 65 years and unless LRTIs can be prevented in these elderly risk-groups, healthcare systems will have difficulties to cope with the disease burden, even more so knowing that almost half of the elderly with a respiratory infection end up in the hospital.^26^ The current focus of health authorities in developed regions is primarily on non-communicable diseases such as heart diseases and prevention of osteoporotic fractures. Substantially less financial resources are spent on prevention and treatment of LRTIs despite their higher incidence, hospitalization rate and overall costs.^27^ As such, governments are required to adapt their health policies and anticipate on the continuously growing LRTI burden amongst the elderly. In doing so, they can avoid their healthcare systems from becoming overburdened, thereby also setting an example for developing nations that eventually will have to deal with their own aging populations. New strategies directed at improving prevention, diagnosis and treatment are therefore required. Increasing vaccine coverage is the most efficient and cost-effective preventive measure but more accurate diagnosis and development of new therapeutic interventions are also crucial to address the emergence of antibiotic resistant bacteria.^28,29^

We have to take into consideration that for study, GDP per capita was chosen as an indicator for economic growth and socioeconomic status. Whereas GDP is frequently used as indicator for a country’s well-being, it does not take into consideration factors such as education and inequalities of income and could therefore be seen as only a rough indicator of a society’s standard of living. To further understand the differential effect of GDP on well-being and respiratory mortality, future studies should also include these socio-economic factors. Also, yearly HAQI values were estimated based on the 5-year values to increase the number of data points to 16. For this estimation we assumed a linear trend between the 5-year values while it fact the values could have been fluctuating. Moreover, the HAQI was calculated based on 32 different diseases, including, but not only limited to, respiratory infections. This makes it complicated to draw solid conclusions as to how exactly improvements in HAQI have been achieved and to what degree LRTIs have been involved.

This study shows that in developing countries the LRTI burden is successfully decreasing, which is likely due to (socio)economic development and improved living standards, as well as international efforts leading to increased vaccine coverage and improved healthcare access and quality. At the same time, however, new challenges arise in wealthier developed nations, where the LRTI burden appears to be growing significantly amongst the aging population. To ensure healthy populations in the long run, health strategies and measures that address this increasing burden require immediate attention.

## Supplemental Material

sj-pdf-1-gph-10.1177_2333794X21989530 – Supplemental material for Lower Respiratory Tract Infection Trends in East and South-East Asia: In the Light of Economic and Health Care Development Click here for additional data file. Supplemental material, sj-pdf-1-gph-10.1177_2333794X21989530 for Lower Respiratory Tract Infection Trends in East and South-East Asia: In the Light of Economic and Health Care Development by Jelle J. Feddema, Anne M. van der Geest, Eric Claassen and Linda H. M. van de Burgwal in Global Pediatric Health
