# Supplementary material for: Lower Respiratory Tract Infection Trends in East and South-East Asia: In the Light of Economic and Health Care Development
Source: Glob Pediatr Health. 2021 Jan 24;8:2333794X21989530. doi: 10.1177/2333794X21989530 (PMC7841237; doi:10.1177/2333794X21989530)
Supplement: sj-pdf-1-gph-10.1177_2333794X21989530 – Supplemental material for Lower Respiratory Tract Infection Trends in East and South-East Asia: In the Light of Economic and Health Care Development [file sj-pdf-1-gph-10.1177_2333794X21989530.pdf]

Lower respiratory tract infection trends in East and South-East Asia: in the light of economic and health care development

Supplementary figures and tables

Classification	GNI per capita	
Lower income economies (LIE)*	< $995	
Lower-middle income economies (LMI)	$996 and $3,895	Cambodia, Indonesia, Laos, Mongolia, Myanmar, Philippines and Vietnam
Upper-middle income economies (UMI)	$3,896 and $12,055	China, Malaysia and Thailand
High income economies (HI)	$12,056 or more	Brunei, Japan, Singapore, South-Korea, and Taiwan

Supplementary Table 1: Classification of countries according to the World Bank Atlas method (19).

*not relevant for this study

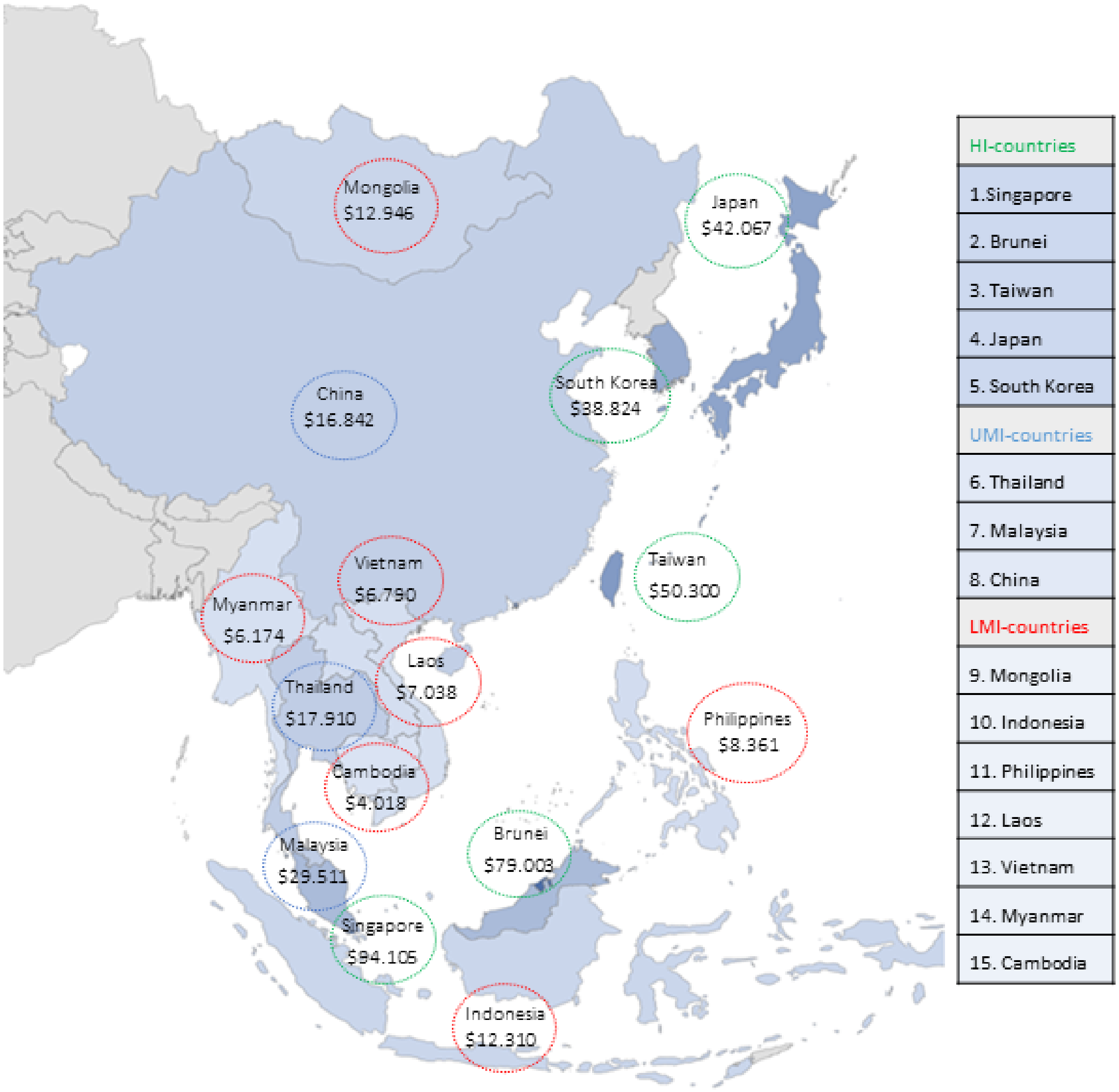

Supplementary Figure 1: Gross domestic product (PPP) per capita per country in 2016 (19).

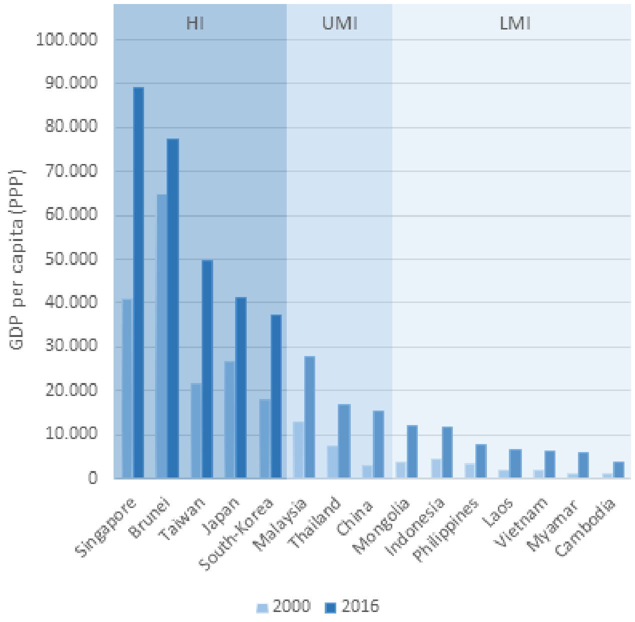

Country	Growth of GDP (2000-2016)	Country classification
Myanmar	+453.20%	LMI
China	+430.18%	UMI
Cambodia	+240.60%	LMI
Laos	+233.01%	LMI
Mongolia	+232.27%	LMI
Vietnam	+210.43%	LMI
Indonesia	+152.65%	LMI
Philippines	+133.30%	LMI
Thailand	+132.87%	UMI
Taiwan	+130.20%	HI
Singapore	+117.71%	HI
Malaysia	+114.56%	UMI
South-Korea	+105.40%	HI
Japan	+53.91	HI
Brunei	+19.21	HI

Supplementary Figure 2: Absolute and relative growth in GDP per capita between 2000 and 2016 for low-middle income countries, upper-middle income countries, and high income countries.

Appendix Correlation Coefficient

	LRTI deaths vs HAQI		LRTI <5 deaths vs HAQI		LRTI deaths growth vs GDP growth		LRTI <5 growth deaths vs GDP growth	
	P value	R	P value	R	P value	R	P value	R
Singapore	<,001 ***	0,9197	,027 *	-0,5504	<,001 ***	0,9557	,007 *	-0,6258
Brunei	<,001 ***	0,8873	<,001 ***	0,9242	<,001 ***	0,8001	<,001 ***	0,9118
Taiwan	<,001 ***	0,9631	,009 **	0,6277	<,001 ***	0,9938	,005 **	0,6520
Japan	<,001 ***	0,9879	<,001 ***	-0,9515	<,001 ***	0,9812	<,001 ***	-0,9610
South-Korea	<,001 ***	0,8706	<,001 ***	-0,9987	<,001 ***	0,9581	<,001 ***	-0,9695
Malaysia	<,001 **	0,9404	,120 ns	-0,4049	<,001 ***	0,9865	,087 ns	-0,4276
Thailand	<,001 ***	0,8067	<,001 ***	-0,8721	<,001 ***	0,9901	<,001 ***	-0,9593
China	<,001 ***	-0,9189	<,001 ***	-0,9799	<,001 ***	-0,7912	<,001 ***	-0,9230
Mongolia	<,001 ***	-0,9897	<,001 ***	-0,9975	<,001 ***	-0,9153	<,001 ***	-0,9364
Indonesia	<,001 ***	-0,9978	<,001 ***	-0,9957	<,001 ***	-0,9967	<,001 ***	-0,9938
Philippines	<,001 ***	0,9451	<,001 ***	-0,8036	<,001 ***	0,8451	<,001 ***	-0,9404
Laos	<,001 ***	-0,9942	<,001 ***	-0,9992	<,001 ***	-0,9606	<,001 ***	-0,9810
Vietnam	<,001 ***	-0,8737	<,001 ***	-0,9824	<,001 ***	-0,9305	<,001 ***	-0,9926
Myanmar	<,001 ***	-0,9955	<,001 ***	-0,9979	<,001 ***	-0,9611	<,001 ***	-0,9730
Cambodia	<,001 ***	-0,9782	<,001 ***	-0,9908	<,001 ***	-0,9320	<,001 ***	-0,9597
